# Age-Adjusted Charlson Comorbidity Index as a Predictor of Clinical Severity in Acute Cholecystitis: A Retrospective Cohort Study

**DOI:** 10.3390/diagnostics16101462

**Published:** 2026-05-11

**Authors:** Marin Davidović, Juraj Kolak, Josip Basić, Anita Zenko Sever, Lucija Brkić, Barbara Kolak, Igor Petrović, Hrvoje Silovski

**Affiliations:** 1 Department of Gastroenteral and Oncologic Surgery, University Hospital Centre Zagreb, 10000 Zagreb, Croatia; marin.davidovic@kbc-zagreb.hr (M.D.); lucija.brkic95@gmail.com (L.B.); 2 Department of Hepatobiliary Surgery and Organ Transplantation, University Hospital Centre Zagreb, 10000 Zagreb, Croatia; igor.petrovic33@gmail.com (I.P.); hrvojesilovski@gmail.com (H.S.); 3 School of Medicine, University of Zagreb, 10000 Zagreb, Croatia; josip.basic000@gmail.com; 4 Department of Pathology, University Hospital Centre Zagreb, 10000 Zagreb, Croatia; anita.zenko@gmail.com; 5 Health Center Zagreb—Center, 10000 Zagreb, Croatia; barbara.kolak1309@gmail.com

**Keywords:** cholecystitis, CCI-Age, surgery

## Abstract

**Background:** Acute cholecystitis is one of the most common acute diseases of the biliary system and a frequent reason for emergency hospital admission and surgical treatment. More severe forms are associated with a higher need for intensive care, more frequent use of open surgery, and less favourable outcomes. Age and chronic comorbidity burden may play an important role in determining disease severity. The age-adjusted Charlson Comorbidity Index (CCI-Age) is a simple and widely used tool for integrated assessment of age and comorbidity burden, but its role in acute cholecystitis is not sufficiently defined. **Methods:** Data for this study were collected by retrospective analysis of hospital records from the hospital information system of the University Hospital Centre Zagreb. Patients treated conservatively were excluded. The association of age and CCI-Age with the severity of the clinical presentation of acute cholecystitis was analysed using dichotomised clinical variables and comparative statistical methods. **Results:** The results showed that age and the presence of chronic diseases were strongly associated with the severity of the clinical presentation of acute cholecystitis. Older patients and those with a greater burden of chronic comorbidities significantly more often developed more severe forms of the disease. Among chronic diseases, peripheral vascular disease, diabetes mellitus, and chronic renal disease showed the strongest association with more severe clinical presentation. **Conclusions:** Age and CCI-Age were strongly associated with the severity of the clinical presentation of acute cholecystitis. CCI-Age may represent a useful tool for early clinical risk stratification and support therapeutic decision making in everyday surgical practice.

## 1. Introduction

Acute cholecystitis is one of the most common acute diseases of the biliary system and a frequent reason for emergency hospital admission and surgical treatment [1,2]. Although in a large number of cases it is a mild form of the disease with a favourable outcome, in a proportion of patients, severe and complicated forms develop, which are associated with an increased incidence of perioperative complications, the need for intensive treatment, and prolonged hospitalisation. Due to the pronounced heterogeneity of the clinical presentation, early assessment of disease severity represents an important element in planning and guiding treatment.

In clinical practice, the assessment of acute cholecystitis severity is based on a combination of clinical signs, laboratory findings, and imaging methods. However, these indicators do not always fully reflect the patient’s overall condition or their ability to respond to acute inflammatory stress. The role of age and the presence of chronic comorbidities are particularly important, as they significantly affect the disease course and treatment outcomes [3]. Older patients and patients with multiple chronic diseases have reduced physiological reserves and are more prone to developing more severe forms of disease and complications.

The age-adjusted Charlson Comorbidity Index (Charlson Comorbidity Index adjusted for age, CCI-Age) is a simple and widely used tool for quantifying comorbidity burden while simultaneously accounting for patient age. Although CCI-Age has been shown to be associated with poorer outcomes in various surgical and medical conditions, its role in assessing the severity of the clinical presentation of acute cholecystitis is not sufficiently well defined. Therefore, the aim of this study was to examine the association of age and the CCI-Age index with the severity of the clinical presentation of acute cholecystitis and to evaluate their clinical value in distinguishing less severe from more severe cases.

## 2. Pathophysiology

### 2.1. Aetiology and Initial Mechanisms

Acute cholecystitis most commonly arises as a consequence of obstruction of the cystic duct by a gallstone, which leads to bile stasis and increased intraluminal pressure in the gallbladder. Bile stasis causes gallbladder distension, reduced perfusion of the wall, and the development of local ischaemia. These processes result in mucosal injury and activation of the inflammatory response, which may initially be sterile, but in later phases, is often complicated by secondary bacterial infection.

Increased intraluminal pressure and ischaemia of the wall promote the release of inflammatory mediators, including cytokines and prostaglandins, which further intensify oedema and infiltration of the gallbladder wall. The intensity of the inflammatory reaction depends on the duration of obstruction, the rate of ischaemia development, and the individual ability of the organism to limit the local inflammatory process.

### 2.2. Progression of Inflammation and Systemic Response

In milder forms of the disease, the inflammatory process remains limited to the mucosa and submucosa of the gallbladder and does not lead to significant systemic disturbances. However, in a certain number of patients, inflammation progresses into deeper layers of the wall, resulting in transmural inflammation. Such progression increases the risk of gangrenous cholecystitis, gallbladder perforation, and peritonitis [1,3].

The development of transmural inflammation and wall necrosis is associated with increased release of pro-inflammatory cytokines, which may lead to systemic inflammatory response syndrome (SIRS), sepsis, and multiorgan failure. Under these circumstances, acute cholecystitis extends beyond local anatomical boundaries and acquires the characteristics of a life-threatening condition requiring intensive treatment and urgent surgical intervention.

### 2.3. Impact of Age and Comorbidities on Disease Course

Patient age plays an important role in the pathophysiological course of acute cholecystitis. Older patients have reduced physiological reserves, a weakened immune response, and a diminished capacity to adapt to acute inflammatory stress. Consequently, their likelihood of containing inflammation at the local level is lower, and the risk of developing severe and complicated forms of the disease is higher.

The presence of chronic comorbidities further worsens the disease course. Diabetes mellitus is associated with impaired microcirculation and weakened immune defence, which favours the development of necrotising forms of cholecystitis. Cardiovascular diseases and chronic heart failure reduce haemodynamic stability and tolerance to inflammatory stress, while chronic renal and pulmonary diseases increase the risk of systemic complications and an unfavourable outcome [4,5].

### 2.4. Clinical Implications

More severe forms of acute cholecystitis are associated with an increased need for treatment in the intensive care unit, more frequent use of an open surgical approach, and a longer duration of hospitalisation. In such patients, perioperative and postoperative complications occur more frequently, and the overall treatment outcome is often less favourable.

Given the key role of age and comorbidities in the pathophysiological mechanisms of disease progression, their integrated consideration represents a rational approach in assessing the severity of the clinical presentation of acute cholecystitis. Indices that combine these factors enable a better assessment of the overall burden on the organism and the potential disease course, thereby facilitating early identification of patients at increased risk and planning of an appropriate therapeutic approach.

## 3. Methodology

### 3.1. Participants

The study included 295 (55.3%) men and 238 (44.7%) women; in total, 218 (40.9%) participants were younger than 65 years, while 315 participants (59.1%) were older than 65 years. The mean age of participants was 65.0 years (SD = 16.4; range 14–96), and the median age was 69 years.

### 3.2. Procedure

Data for this study were collected by retrospective analysis of hospital records from the hospital information system of the University Hospital Centre Zagreb. Patients treated conservatively were excluded from the study. The analysis included patients treated for acute cholecystitis in the period from March 2021 to March 2025. Demographic data, clinical parameters, laboratory findings, and data on the treatment course and disease outcome were collected from the hospital information system. All collected data were processed in an anonymised manner without the possibility of identifying individual patients.

The study was conducted in accordance with ethical principles and applicable personal data protection regulations. As this was a retrospective analysis of previously collected data, there were no additional interventions and no direct contact with patients for the purposes of this study.

### 3.3. Operationalisation of Variables

For the purpose of testing the study hypotheses, selected clinical and demographic parameters were recoded into dichotomous variables. This enabled clearer differentiation between less severe and more severe cases of cholecystitis and the application of appropriate statistical tests.

Age was categorised into two groups: younger patients (<65 years) and older patients (≥65 years). The threshold of 65 years was selected due to the clinical and epidemiological importance of age in the development of complications and treatment outcomes.

CCI-Age (Charlson Comorbidity Index adjusted for age) was recoded so that values <6 were assigned to the less severe group, while values ≥6 indicated increased risk and were assigned to the more severe group. This cut-off value was chosen based on previous studies in which CCI ≥ 6 is considered a predictor of poorer outcome [4,6,7].

CRP (C-reactive protein) was classified based on a cut-off value of 100 mg/L. Patients with CRP values <100 mg/L were assigned to the less severe group, whereas patients with CRP ≥ 100 mg/L were assigned to the more severe group. This threshold is based on clinical guidelines that associate elevated CRP values with more pronounced inflammation and a more complex surgical course [8,9].

Case severity was defined as a composite variable with two categories: less severe and more severe cases. The more severe group included all patients who met at least one of the following criteria:

- CRP value ≥100 mg/L;
- CCI-Age value ≥6;
- Need for laparotomy;
- Admission to the intensive care unit (ICU).

Patients who did not meet any of the above criteria were classified as having a less severe clinical presentation.

In this way, the operationalised severity variable enabled a comprehensive assessment of patients’ clinical status by taking into account multiple aspects of the disease and its treatment.

Descriptive statistics for the variables “Urgency” and “Reoperation” were performed on the non-recoded variables, where the number 1 indicated “No” and the number 2 indicated “Yes”. All other variables were recoded so that lower values and more favourable (less severe) outcomes were coded as 0, and higher values and less favourable (more severe) outcomes as 1, while for the remaining dichotomous responses, the coding No = 0, Yes = 1 was applied.

This composite definition was chosen to capture multiple dimensions of disease severity and treatment burden; however, the inclusion of CCI-Age within the severity construct may introduce conceptual overlap when interpreting its association with clinical severity.

## 4. Results

Elevated C-reactive protein (CRP) values were recorded in 50.7% of patients and a high comorbidity burden (CCI-Age ≥ 6) in 34.3% of patients. When the overall composite severity criterion was considered, 68.1% of patients were classified as more severe cases (Table 1).

The largest proportion of patients underwent emergency surgery (95.5%), whereas reoperation was performed in a small number of patients (0.6%). According to the laparoscopic surgery criterion, 34.1% of patients were classified as more severe cases, and 20.1% of patients were treated in the intensive care unit (ICU).

All patients in the less severe group had CCI-Age < 6, whereas in the more severe group, almost half had CCI-Age < 6 (49.6%), and the other half had CCI-Age ≥ 6 (50.4%). Overall, 37.0% of all patients had elevated CCI-Age values (≥6) (Table 2).

The analysis showed a statistically significant association between all examined predictors and clinical severity (*p* < 0.001). C-reactive protein (CRP) had the highest χ^2^ and Φ values (χ^2^ = 205.43, Φ = 0.67). This was followed by CCI-Age ≥ 6 (χ^2^ = 104.90, Φ = 0.46) and laparotomy (χ^2^ = 103.73, Φ = 0.46), while intensive care unit (ICU) admission had the lowest values (χ^2^ = 44.08, Φ = 0.31) (Table 3).

The analysis showed that several CCI-Age components were significantly associated with a less favourable (more severe) clinical outcome. The strongest associations were found for peripheral vascular disease (χ^2^ = 99.61, *p* < 0.001, Φ = 0.45) and diabetes mellitus (χ^2^ = 71.74, *p* < 0.001, Φ = 0.38). Significant associations were also observed for severe renal disease, myocardial infarction, heart failure, and any malignancy (all *p* < 0.001). Weaker but still significant associations were found for dementia, chronic pulmonary disease, peptic ulcer disease, and diabetes with chronic complications. No significant associations were observed for mild liver disease, hemiplegia, leukaemia, lymphoma, or moderate or severe liver disease. There were no patients with AIDS in the sample (Table 4).

## 5. Discussion

The results of this study showed a clear and statistically significant association between age, the CCI-Age index, and the severity of the clinical presentation of acute cholecystitis. Older patients and patients with higher CCI-Age values were significantly more likely to belong to the group of more severe clinical cases, which confirms the importance of these factors in assessing risk and disease course. The obtained findings are consistent with previous knowledge that age and comorbidities play a key role in the development of complications and unfavourable outcomes in acute surgical conditions [4,4,5,7,10,11].

These findings are consistent with previously published data showing that advanced age and greater comorbidity burden are associated with worse outcomes in acute surgical conditions. Earlier studies have demonstrated that the Charlson Comorbidity Index and its age-adjusted form may be useful for outcome assessment in emergency general surgery, while studies in acute cholecystitis have also suggested that higher CCI-Age values are associated with a less favourable clinical course. In that context, our results add further support to the clinical relevance of CCI-Age, specifically in patients with acute cholecystitis, by showing a strong association between comorbidity burden and more severe clinical presentation.

A particularly notable finding of this study was the strong association between CCI-Age ≥ 6 and more severe clinical presentation. However, this finding should be interpreted with caution, as the composite severity variable included CCI-Age ≥ 6 among its defining criteria. Therefore, although the observed association remains clinically relevant, the possibility of conceptual overlap and overestimation of discriminatory performance must be acknowledged.

These findings suggest that CCI-Age may be clinically useful already at hospital admission, particularly for early risk stratification and identification of patients who may require closer monitoring or more intensive perioperative management. In this context, CCI-Age may complement standard clinical, laboratory, and imaging assessment in everyday surgical decision making.

In the analysis of predictors of the severity of the clinical presentation, laboratory parameters of inflammation may show high values of statistical association, but their interpretation must be cautious. Laboratory findings such as elevated values of inflammatory markers reflect the current state of the disease, whereas the CCI-Age index integrates long-term overall physiological burden through age and chronic comorbidities [10,11]. Therefore, although certain laboratory parameters may show a stronger statistical association, the CCI-Age index has greater clinical value in assessing overall patient risk and the potential course of disease [8,9].

A more detailed analysis of individual components of the CCI-Age index showed that not all chronic diseases contribute equally to the development of a more severe clinical presentation of acute cholecystitis. The most pronounced association with a more severe clinical presentation was observed for peripheral vascular disease (Φ = 0.45), which indicates an important role of microcirculatory disturbances and reduced perfusion reserve in disease progression. A significant association was also shown for diabetes mellitus (Φ = 0.38), especially in the context of impaired immune response and healing, as well as severe renal disease, myocardial infarction, and heart failure, which further limit the patient’s haemodynamic stability [12].

In contrast, certain components of the CCI-Age index, such as mild liver disease, hemiplegia, and haematological malignancies, did not show a significant association with the severity of the clinical presentation, which confirms that the overall CCI-Age index, as well as the comorbidity profile, have an important role in risk assessment.

The obtained results can be explained by pathophysiological mechanisms associated with ageing and the presence of chronic diseases. Older patients have reduced physiological reserves and a weakened immune response, while comorbidities such as diabetes mellitus and cardiovascular and renal diseases further limit the body’s ability to respond to acute inflammatory stress. Under such circumstances, the inflammatory process more often extends beyond local boundaries and leads to the development of severe and complicated forms of acute cholecystitis, which is consistent with the findings of this study.

The clinical applicability of the CCI-Age index represents one of the important advantages of this tool. It is a simple and readily available indicator that can be calculated on admission, without the need for additional diagnostic procedures. In everyday clinical practice, the CCI-Age index may serve as a useful tool for early patient stratification, identification of those at increased risk, and planning treatment intensity and the surgical approach [6,13].

The limitations of this study include its retrospective design, which entails inherent limitations related to the availability and completeness of data. Furthermore, the study was conducted in a single institution, which may limit the generalisability of the results. An additional methodological limitation is that the composite clinical severity variable included CCI-Age ≥ 6 as one of its defining criteria, while CCI-Age was also analysed in relation to severity. This may have introduced conceptual overlap and contributed to an overestimation of the observed association. In addition, the use of dichotomised variables, although clinically practical, may have resulted in some loss of information and reduced statistical sensitivity. Finally, the absence of adjusted multivariable analyses limits the ability to assess the independent contribution of individual predictors. Despite these limitations, the relatively large sample and clearly defined disease-severity criteria contribute to the reliability of the obtained findings.

In conclusion, the results of this study confirm that age and the CCI-Age index are strongly associated with the severity of the clinical presentation of acute cholecystitis. The findings suggest that CCI-Age may be a useful tool for early clinical risk stratification and therapeutic decision making in patients with acute cholecystitis. However, these results should be interpreted in light of the methodological limitations of the composite severity definition used in this study and require further validation in prospective studies.

## 6. Conclusions

The results of this study showed that age and the presence of chronic diseases are strongly associated with the severity of the clinical presentation of acute cholecystitis. Older patients and patients with a greater burden of chronic comorbidities more often developed more severe forms of the disease, highlighting the importance of comprehensive patient assessment on admission. Among chronic diseases, peripheral vascular disease, diabetes mellitus, and chronic renal disease showed the strongest association with more severe clinical presentation.

The age-adjusted Charlson Comorbidity Index (CCI-Age) may represent a useful tool for the integrated assessment of age and chronic disease burden and may facilitate the early identification of patients at increased risk. However, because of the methodological limitations of the composite severity definition used in this study, further prospective validation is needed before broader clinical application.

## Figures and Tables

**Table 1:** Descriptive statistics for surgical-severity criteria.

Variable	Category	*N* (%)	Mean	Median	Mode	SD
**Urgency**	Yes	509 (95.5%)	1.95	2	2	0.208
**Urgency**	No	24 (4.5%)				
**Reoperation**	Yes	3 (0.6%)	1.01	1	1	0.075
**Reoperation**	No	530 (99.4%)				
**Laparoscopy**	No	310 (58.2%)	0.370	0	0	0.483
**Laparoscopy**	Yes	182 (34.1%)				
**ICU**	No	362 (67.9%)	0.228	0	0	0.420
**ICU**	Yes	107 (20.1%)				
**CRP**	Less severe	188 (35.3%)	0.590	1	1	0.492
**CRP**	More severe	270 (50.7%)				
**CCI-Age**	Less severe	311 (58.3%)	0.370	0	0	0.483
**CCI-Age**	More severe	183 (34.3%)				
**Overall severity (composite)**	Less severe	131 (24.6%)	0.735	1	1	0.424
**Overall severity (composite)**	More severe	363 (68.1%)				

**Table 2:** Relationship between CCI-Age and overall clinical severity.

Clinical Severity	CCI-Age < 6 *N* (%)	CCI-Age ≥ 6 *N* (%)	Total *N* (%)
**Less severe cases**	131 (100.0%)	0 (0.0%)	131 (100%)
**More severe cases**	180 (49.6%)	183 (50.4%)	363 (100%)
**Total**	311 (63.0%)	183 (37.0%)	494 (100%)

Note: χ^2^ = 104.9, *p* < 0.001, Φ = 0.46.

**Table 3:** Association of predictors with clinical severity.

Predictor	χ^2^ (1)	*p*	Φ
**CRP**	205.43	<0.001	0.67
**CCI-Age ≥ 6**	104.90	<0.001	0.46
**ICU**	44.08	<0.001	0.31
**Laparotomy**	103.73	<0.001	0.46

**Table 4:** Contribution of individual CCI-Age components to clinical severity.

CCI Component	χ^2^ (1)	*p*	Φ
**Myocardial infarction (MI)**	54.26	<0.001	0.33
**Congestive heart failure (CHF)**	51.45	<0.001	0.32
**Peripheral vascular disease (PD)**	99.61	<0.001	0.45
**Cerebrovascular disease (CVI)**	49.38	<0.001	0.32
**Dementia**	9.67	0.002	0.14
**Chronic pulmonary disease (CPD)**	13.80	<0.001	0.17
**Rheumatic disease (RD)**	0.07	0.788	0.01
**Peptic ulcer disease**	23.70	<0.001	0.22
**Mild liver disease**	3.44	0.06	0.08
**Diabetes mellitus (DM)**	71.74	<0.001	0.38
**Diabetes with chronic complications**	10.32	0.006	0.15
**Hemiplegia**	3.56	0.168	0.09
**Renal disease**	55.21	<0.001	0.34
**Any malignancy**	50.83	<0.001	0.32
**Leukaemia**	1.70	0.192	0.06
**Lymphoma**	0.00	0.982	0.00
**Moderate or severe liver disease**	4.19	0.123	0.09
**Metastatic solid tumour**	22.69	<0.001	0.21
**AIDS** *	/	/	/

* Note: There were no patients with AIDS in the sample.

## Data Availability

The data presented in this study are not publicly available due to privacy and ethical restrictions.

## Abbreviations

The following abbreviations are used in this manuscript:

AIDS	Acquired immunodeficiency syndrome
CCI	Charlson Comorbidity Index
CCI-Age	Age-adjusted Charlson Comorbidity Index
CHF	Congestive heart failure
CPD	Chronic pulmonary disease
CRP	C-reactive protein
CVI	Cerebrovascular disease
DM	Diabetes mellitus
ICU	Intensive care unit
MI	Myocardial infarction
PD	Peripheral vascular disease
RD	Rheumatic disease
SIRS	Systemic inflammatory response syndrome
SD	Standard deviation
